# Diphtheria

**Published:** 1889-05

**Authors:** Jno. Preston

**Affiliations:** Austin, Texas, President Travis County Medical Society, and Ist Ass’t Physician State Lunatic Asylum


DANIEL'S

### Texas Medical Journal.

A Representative Organ of the Medical Profession, and an Exponent of Rational Medicine; devoted to the Organization, Advancement and Elevation of the Profession in Texas.

PUBLISHED MONTHLY.—SUBSCRIPTION $2.00 A YEAR.

Vol. IV. AUSTIN, MAY, 1889. No. 11.

### Original Articles.

CONTRIBUTED EXCLUSIVELY TO THIS JOURNAL.

*The Articles in this Department are accepted and published with the understanding that we are not responsible for, nor do we indorse the views and opinions of the writers by so doing.*

### DIPHTHERIA.

BY JNO. PRESTON, M. D., PRESIDENT TRAVIS COUNTY MEDICAL SOCIETY, AND 1ST ASS'T PHYSICIAN STATE LUNATIC ASYLUM.

Read before Travis County Medical Society and published in Daniel's Texas Medical Journal by vote of Committee.

THE history of diphtheria extends back to a remote period. It was recognized in the time of Hypocrites; was described by Homer, and has been treated of by numerous writers since, under various names—such as ——— Egyptiaçum, cynanche maligna, morbus suffocans, epidemic croup, diphtheritis, etc. Britonneau in 1821, gave the disease the name of diphtheria, and the modern history of the affection begins with his papers on the subject. He asserted the identity of diphtheria and croup and stated that the membrane found in the larynx was but a continuation of that discovered in the pharynx. From this time on many writers have appeared and given their views. The main points of difference between them being, whether diphtheria is a constitutional disease with local manifestations, or a local inflammation with constitutional disturbances; and whether diphtheria and croup are distinct diseases. To this day the matter is not settled, there existing good authority on each side of the question.

Diphtheria is a contagious and infectious disease, and occurs generally as an epidemic. Its prodromic symptoms do not differ in a marked degree from those of many other affections. The patient complaining of dullness, chilly sensations, has slight fever, is indisposed and experiences difficulty in swallowing. The mucous membrane is red and swollen. As the disease progresses a false membrane will be observed beginning in small patches and rapidly extending until it covers all or a greater part of the fauces. This membrane may, however, be confined to the tonsils, palate or nares or it may involve the whole of the pharynx and extend to the larynx and trachea. When localized in the larynx in has been termed diphtheria croup. From the first observance of the deposit it requires about 24 hours for the patches to coalesce and form a complete membrane. This membrane is characteristic of the disease and is regarded as pathognomonic. It may be simply an exudation on the mucous membrane and lay loosely upon it, or it may involve the mucous membrane and subjacent tissues. In the latter case there will generally occur sloughing of the soft tissues.

In a typical case of diphtheria the following conditions are presented :

1st Stage. The patient complains of indisposition to exertion, loss of appetite, soreness about the throat, chilliness and nausea. There is generally a rise of temperature which may reach 105 F.

The neck becomes stiff from swelling of the glands and there is difficult deglutition. The mucous membrane covering the tonsils uvula and fauces is red and swollen.

2nd Stage. In the 2nd stage a grayish yellow secretion will begin to be deposited on the tonsils and will extend to different parts of the mucous membrane.

These deposits spread until they extend over the whole of the pharynx and form a pseudo-membrane, which, when removed, represents an exact cost of the parts affected. The fever diminishes generally when the exudation appears. The breath becomes foul from the morbid secretions and their decomposition. The pulse becomes weak and the cardiac impulse less distinct. The urine is high colored and reduced in quantity. If the exudation should extend to the larynx and trachea, the breathing will be greatly interfered with, and there will be croupous cough and a wheezing sound as the air is expelled from the lungs. Coma may follow from improper oxigenation of the blood and the patient may die. The air passages may become permanently involved. Then there is a fetid discharge from the nose as the exudation is formed. Sometimes epistaxis is a prominent symptom. The eustachian tube may be seat of the mobid process, then the ear is affected. The conjuctiva may also become inflamed and the characteristic deposit be seen there. In fact, any exposed mucous membrane may become affected and even the skin, when deprived of the epidermis.

3rd Stage.—During this stage the patient begins to improve. The false membrane becomes loose and is cast off in fragments, anorexia disappears; the swelling ceases, the pulse increases in volume, the breathing becomes easy, and the patient begins to look brighter and more cheerful. This stage commences about the beginning of the second week. Then there remains one great danger. and that is blood poisoning. Should this occur there will be a rapid return of fever and all the symptoms peculiar to septicæmia. If the heart is much affected, we may have syncope from heart failure. In mild cases the symptoms presented above may be very much modified. The false membrane may be absent, as there may be only slight deposits at different points. Such cases may be coufounded with acute tonsilitis, pharyngitis or laryngitis. It is often difficult to make a differential diagnosis between diphtheria and scarlatina. The absence of the false membrane and red appearance of the skin in the latter are to be taken into consideration in making a diagnosis. From acute laryngitis, unless the fact is settled that an epidemic of diphtheria exists, it is almost impossible to make a diagnosis in the early stages.

PROGNOSIS.—In every case the prognosis must be guarded. It must be based upon the symptoms peculiar to the individual case. If serious complications exist, as, a weakened heart, pneumonia or renal trouble, or if blood-poisoning should occur, the prognosis is unfavorable. Should none of these complications occur, and the air passages not become involved, and the patient enter the stage of convalescence well preserved in strength, with a return of appetite, no diarrhea, nausea, hemorrhages, or other serious complications, a favorable prognosis may be given.

ETIOLOGY.—Diphtheria occurs generally in the winter months. A moist condition of the atmosphere seems to favor its production. It is a disease peculiar to children, though those of mature years are not exempt from its ravages. The rich and the poor are attacked alike, though poor hygienic surroundings and want of proper nutrition seem to favor its spread. Recent investigators have, as in most other epidemic diseases, discovered bacteria peculiar to this disease. I will not undertake to describe the peculiar shape or give the exact dimensions of these microscopic organisms. I have never seen them, though I do not doubt their existence. Diphtheria is contagious and infectious, usually occurring as an epidemic.

TREATMENT.—The first object in the treatment of diphtheria is to use every means in our power to sustain the strength of the patient. To this end, the most nutritous and easily digested diet should be administered at short intervals. Milk, beef tea or essence, and eggs, combined with stimulants, may be mentioned under this head.

The temperature of the room should be kept as near 65 F. as possible, and free ventilation secured. In this climate the windows on the south side of the house can be left open the greater part of the time, even in winter. An open fire favors perfect ventilation. As to disinfection, a strict adherence to the rules laid down by the National Board of Health should be observed.

Fumigation of the rooms with sulphur, and the boiling of the exposed clothing in water containing sulphate of zinc or corrosive sublimate, are to be recommended. Sulphate of iron solution is perhaps the best disinfectant for the chamber vessels. It should be used bountifully about the premises, and especially the privies.

INTERNAL TREATMENT—Jacobi recommends the chlorate of potassa as a preventive remedy. Steam inhalations of pure or medicated waters are highly praised by some authors. The atomizer is probably the best means of applying remedies direct to the affected surfaces. A solution of salicylate of sodium, chloride of sodium, carbolic acid or listerine can be conveniently employed by this means. In the steam inhalations, turpentine or lime is often added to the water with good effect. They assist in allaying the irritation and in loosing the false membrane.

When the patches of membrane begin to form, the application of salicylic acid is favorably spoken of by recent writers, as being a remedy that retards its progress. It also destroys the fetid odor of the breath.

The secretions should be regulated. The administration of an occasional dose of calomel seems to meet this indication better than other drugs. It is said to have a special effect upon the deposit. Boric acid, sulphite of sodium, chlorate of potassa, sulphate of zinc, and tannin and glycerine solution, are used freely as gargles, and often with marked benefit. Quinine, salicylate of sodium and anti-pyrin must be employed should the temperature rise above 103 F. Tonic doses of quinine are admissible during the whole course of the disease.

The membrane should never be removed by force, unless greatly interfering with respiration. The forcible tearing off of the membrane often causes an irritation of the parts and leaves a raw surface for the reception of the poison, thereby favoring a second attack of blood poisoning. The application of ice to the neck and swallowing small pieces of ice are very grateful to the patient, and seem to diminish the swelling of the mucous membrane. Should the disease attack the eye, cold applications of boric acid or sulphate of zinc solution must be used. The other eye should be protected.

Blood poisoning should be treated according to the principles observed in the management of that complication. Paralysis should be treated by rest, tonics and strychnia. If there exists great distress in breathing from diphtheric croup, placing the patient in imminent danger of death, tracheotomy should be performed. This should not be delayed too long, as the vital powers may become so lowered as to render the patient unable to sustain the shock of the operation.

Intubation in extreme cases has been practiced recently with good results. I have no practical knowledge of this treatment.

In this disease, as in all others, no treatment can be laid down as suitable to every case. The physician must watch the symptoms and apply such remedial agents as in his judgment are indicated. Those that I have mentioned will form a list, I believe, from which we can select remedies most applicable to the treatment of this most terrific scourge.

